# Supplementary material for: Nanoscale magnonic Fabry-Pérot resonator for low-loss spin-wave manipulation
Source: Nat Commun. 2021 Apr 16;12:2293. doi: 10.1038/s41467-021-22520-6 (PMC8052321; doi:10.1038/s41467-021-22520-6)
Supplement: Supplementary file 1 — Supplementary Information [file 41467_2021_22520_MOESM1_ESM.pdf]

Nanoscale magnonic Fabry-Pérot resonator for low-loss spin-wave manipulation

Huajun Qin[1*], Rasmus B. Holländer[1], Lukáš Flajšman[1], Felix Hermann[1,2], Rouven Dreyer[3], Georg Woltersdorf[3], & Sebastiaan van Dijken[1*]

[1]*NanoSpin, Department of Applied Physics, Aalto University School of Science, FI-00076 Aalto, Finland*

[2]*Physikalisches Institut, Karlsruhe Institute of Technology, Wolfgang-Gaede-Str. 1, Karlsruhe D-76131, Germany*

[3]*Institute of Physics, Martin Luther University Halle-Wittenberg, 06120 Halle, Germany*

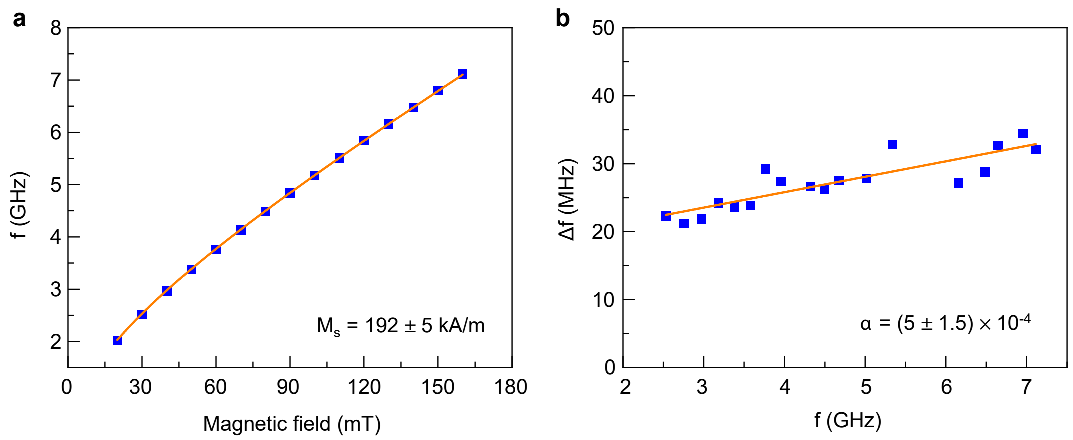

Supplementary Figure 1. **a** Frequency of the YIG FMR mode as a function of magnetic bias field measured by placing a 100-nm-thick YIG film face-down onto a coplanar waveguide. The Kittel-formula fit (orange line) of the experimental data gives a saturation magnetization M_s = (1.92 $\pm$ 0.05) $\times 10^5$ A/m. **b** Linewidth of the YIG FMR mode as a function of frequency. A linear fit to the data using $\Delta f = 2\alpha f + v_\mathrm{g}\Delta k$ yields a Gilbert damping constant α = (5 $\pm$ 1.5) $\times 10^{-4}$.

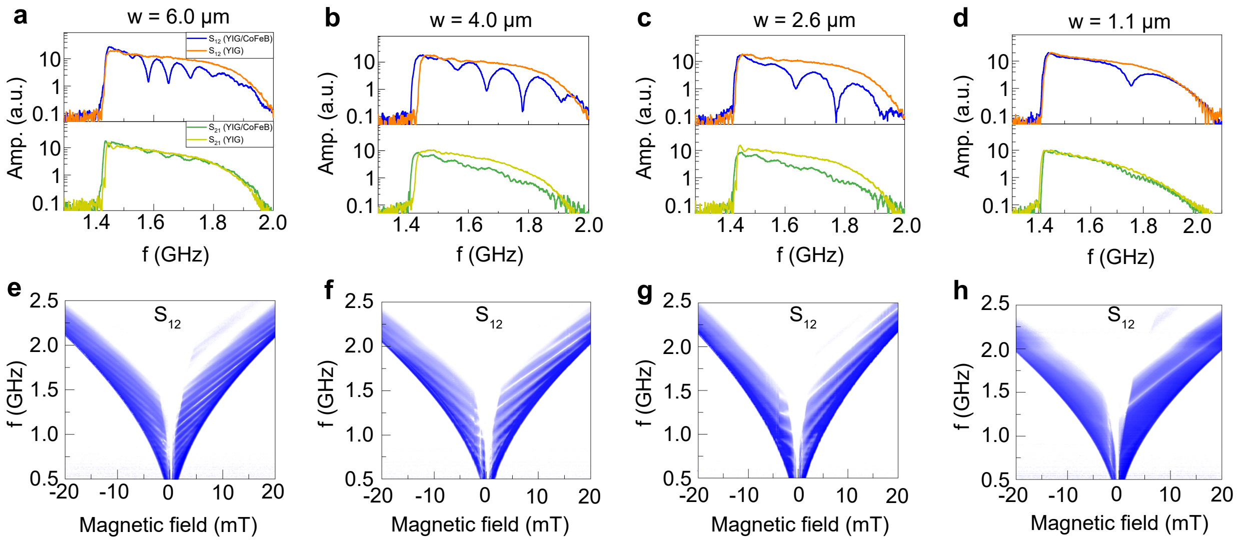

Supplementary Figure 2. **a-d** Spin-wave transmission spectra (amplitude of S_{12} and S_{21}) recorded on a 100-nm-thick YIG film with a CoFeB stripe. The width of the CoFeB stripe is 6.0 μm, 4.0 μm, 2.6 μm, and 1.1 μm, respectively. $\mu_0 H_{\text{ext}} = +10$ mT. Spectra for an uncovered YIG film are shown as reference. **e-h** Contour plots of the S_{12} amplitude as a function of magnetic field for the same samples.

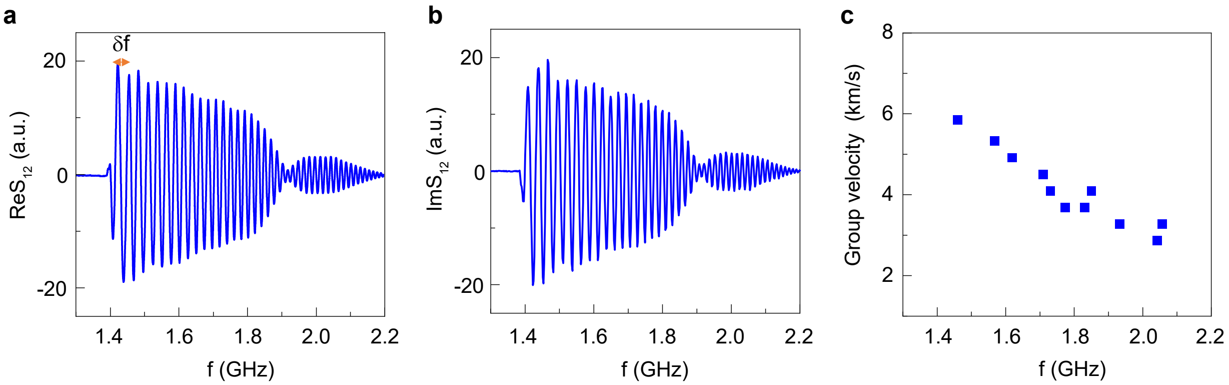

Supplementary Figure 3. **a,b** Real and imaginary part of scattering parameter S_{12} recorded on a 100-nm-thick YIG film with a 730-nm-wide CoFeB stripe. $H_{\mathrm{ext}} = +10$ mT. **c** Corresponding group velocity υ_{g} of propagating spin waves, as extracted using $\upsilon_{\mathrm{g}} = \delta f \times s$. Here, δf is the frequency separation between two maxima or minima in the transmission spectrum (see arrow in **a**) and $s = 200$ μm is the spin-wave propagation distance (separation of microwave antennas).

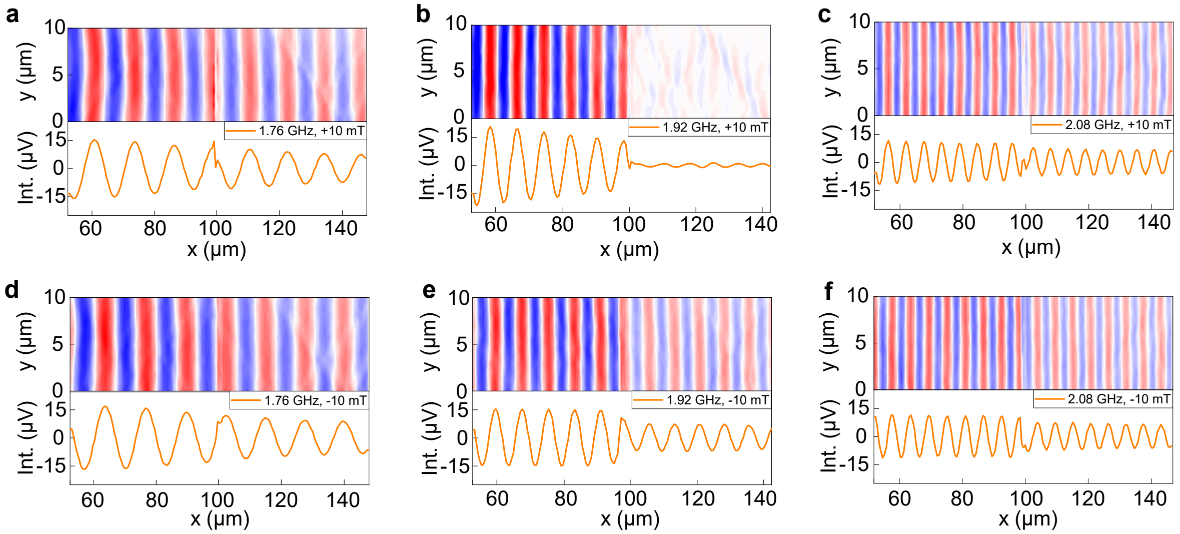

Supplementary Figure 4. **a-c** TR-MOKE microscopy maps and line profiles of propagating spin waves in a 100-nm-thick YIG film with a 730-nm-wide CoFeB stripe. The excitation frequency is 1.76 GHz (**a**), 1.92 GHz (**b**) and 2.08 GHz (**c**), respectively. $\mu_0 H_{\text{ext}} = +10$ mT. **d-f** TR-MOKE microscopy maps and line profiles recorded on the same sample as in (**a-c**), but with $\mu_0 H_{\text{ext}} = -10$ mT. At the gap frequency (1.92 GHz), switching the magnetic bias field from $+10$ mT to -10 mT increases the transmission of spin waves across the YIG/CoFeB bilayer region. Consequently, the resonance effect producing minimum transmission is nonreciprocal. This result agrees with the broadband spin-wave transmission spectra of Figs. 1b,c in the main manuscript. At allowed frequencies (1.76 GHz and 2.08 GHz), spin waves efficiently propagate across the bilayer region for $\mu_0 H_{\text{ext}} = +10$ mT and $\mu_0 H_{\text{ext}} = -10$ mT.

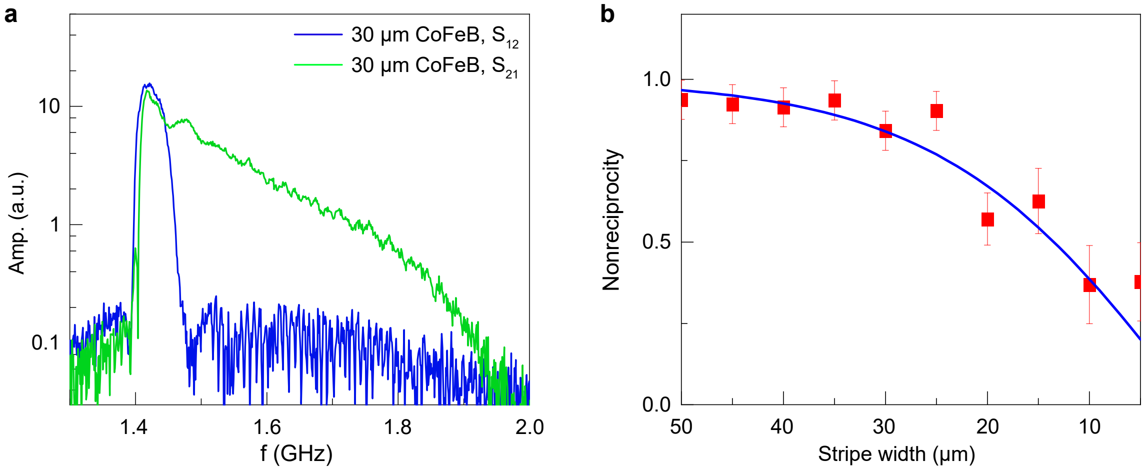

Supplementary Figure 5. **a** Spin-wave transmission spectra (amplitude of S_{12} and S_{21}) recorded on a 100-nm-thick YIG film with a 30-μm-wide CoFeB stripe. $\mu_0 H_{\text{ext}} = +10$ mT. The S_{12} signal is fully suppressed over a broad frequency range (1.48 GHz - 2.0 GHz), whereas S_{21} is large in the entire excitation range. **b** Experimentally derived nonreciprocity coefficient defined as $\eta = |S_{21} - S_{12}|/|S_{21} + S_{12}|$ at 1.58 GHz for a 100-nm-thick YIG film with single CoFeB stripes of varying width. The line is a fit to the data using $\eta = (\exp(-w/l_{\text{d},\lambda_2}) - \exp(-w/l_{\text{d},\lambda_3}))/(\exp(-w/l_{\text{d},\lambda_2}) + \exp(-w/l_{\text{d},\lambda_3}))$. Using $l_{\text{d},\lambda_2} = 42$ μm derived from TR-MOKE microscopy data (Supplementary Fig. 6), we extract $l_{\text{d},\lambda_3} = 10$ μm for the decay length of λ_3 waves.

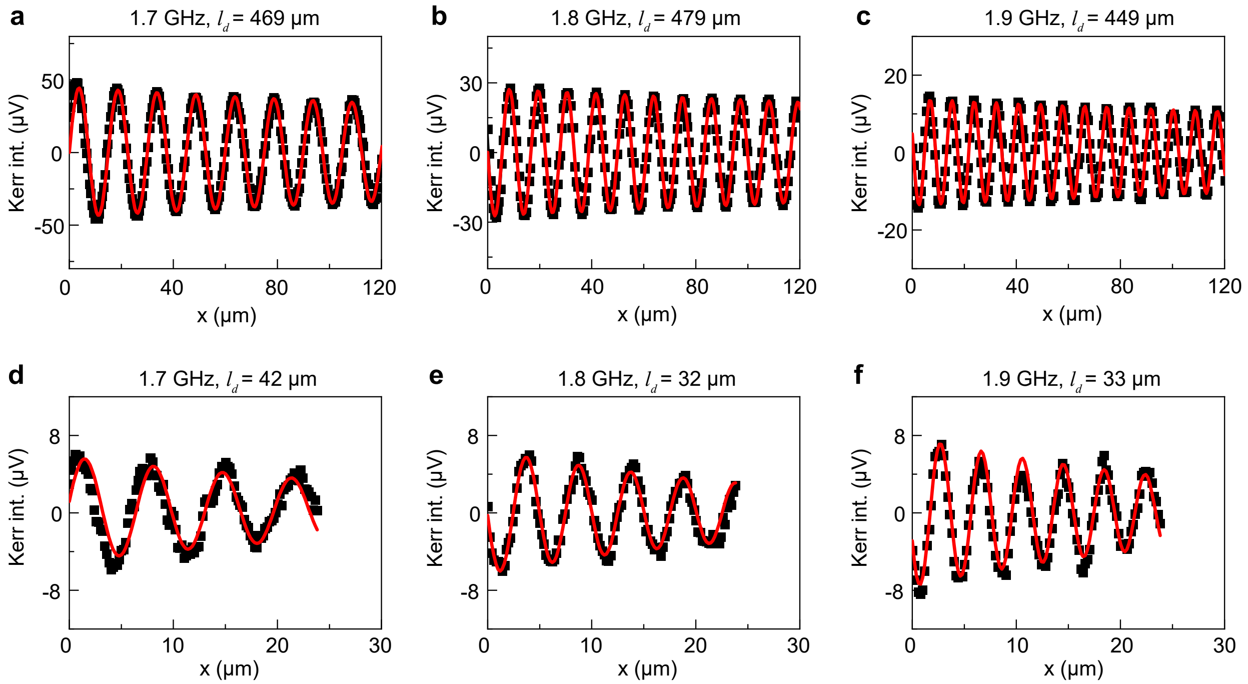

Supplementary Figure 6. **a-f** TR-MOKE microscopy line profiles for λ_1 waves in an uncoverd 100-nm-thick YIG film (**a-c**) and λ_2 waves in a 100 nm YIG/50 nm CoFeB bilayer (**d-f**) at three frequencies. The decay length of the propagating modes (l_d) are extracted by fitting the data to $C\exp(-x/l_\mathrm{d})\sin(2\pi x/\lambda+\phi)$. The extracted decay lengths are given above the graphs.

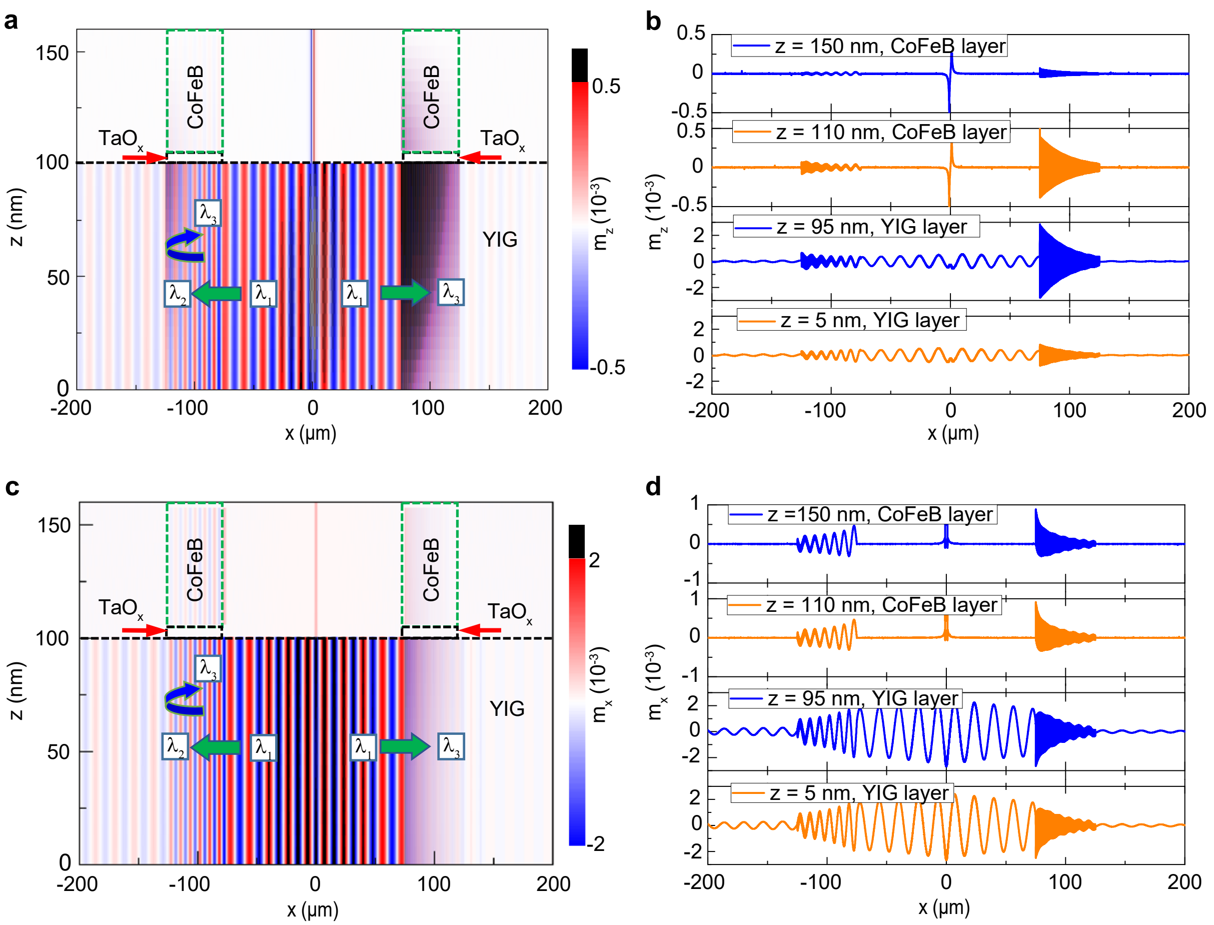

Supplementary Figure 7. **a,c** Simulated spatial distribution of m_z and m_x in a 100-nm-thick YIG film with two 50-nm-thick CoFeB stripes that are separated from YIG by 5-nm-thick non-magnetic spacers (representing TaO$_x$ in the experiments). The CoFeB stripes are 50 μm wide. $H_{\text{ext}} = +10$ mT and $f = 1.70$ GHz. Spin waves with wavelength λ_1 are excited continuously at $x = 0$. The YIG/CoFeB bilayer regions are located at $-75\ \mu\text{m} \geq x \geq -125\ \mu\text{m}$ and $75\ \mu\text{m} \leq x \leq 125\ \mu\text{m}$. At the edges of the YIG/CoFeB bilayer, the incoming λ_1 spin waves convert to λ_2 and λ_3 modes. The long-wavelength λ_1 and λ_2 modes are approximately uniform across the YIG film thickness, whereas m_z of the short-wavelength λ_3 mode decays gradually from the top surface.

Panels **b,d** show corresponding line profiles extracted at $z = 150$ nm (CoFeB), $z = 110$ nm (CoFeB), $z = 95$ nm (YIG), and $z = 5$ nm (YIG). From the line profiles, we extract transmission coefficients $t_{12} = 1$ and $t_{21} = 0.5$. The λ_1 and λ_2 spin waves are elliptical along x ($\epsilon = 0.8$). The λ_3 mode is elliptical along z ($\epsilon = 0.3$) at $z = 95$ nm and along x ($\epsilon = 0.5$) at $z = 5$ nm.

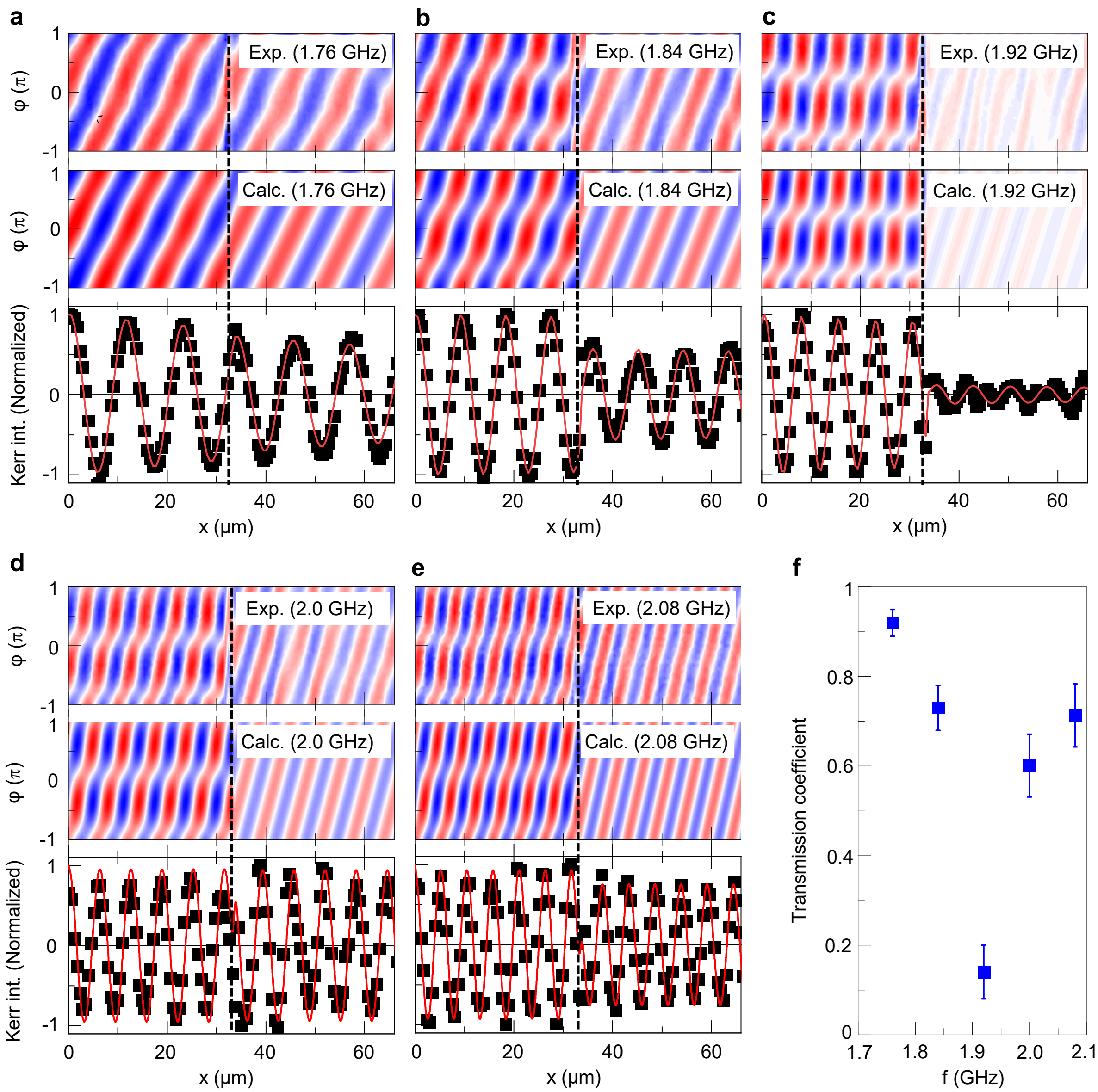

Supplementary Figure 8. **a-e** Phase-resolved TR-MOKE microscopy maps (top panels), calculated 2D maps (middle panels), and fits (red lines) to selected experimental line profiles (symbols) (bottom panels) for a 100-nm-thick YIG film with a 730-nm-wide CoFeB stripe at five different frequencies. The dashed lines indicate the location of the CoFeB stripe. In the calculations, an incoming wave, reflected wave, and transmitted wave are considered, but wave conversions and internal reflections inside the nanoscale resonator are omitted.

In other words, the model calculates the total reflection and transmission of the resonator. To reproduce the experimental data, we divide the measurement space into a region before the resonator ($x = 0 \ldots x_{\mathrm{res}}$), where the incident wave A_1 with amplitude A_{in} at $x = 0$ and the reflected wave from the resonator A_2 with amplitude A_{R} at x_{res} sum up, and a region after the resonator ($x = x_{\mathrm{CoFeB}} \ldots x_{\mathrm{max}}$), into which a wave A_3 transmits with amplitude A_{T}. The spatial evolutions of the three waves $A_1,\ A_2,\ A_3$ are then given by

$$A_1\left(x = 0 \ldots x_{\mathrm{res}}\right) = A_{\mathrm{in}} \mathrm{e}^{-\frac{x}{l_{\mathrm{d}}}} \cos\left(2\pi \frac{x}{\lambda_1} + \phi_{\mathrm{in}} + \Delta\phi_{\mathrm{in}}\right),$$

$$A_2\left(x = 0 \ldots x_{\mathrm{res}}\right) = A_{\mathrm{R}} \mathrm{e}^{\frac{x - x_{\mathrm{res}}}{l_{\mathrm{d}}}} \cos\left(2\pi \frac{-x + x_{\mathrm{res}}}{\lambda_1} + \phi_{\mathrm{A}_1}\left(x_{\mathrm{res}}\right) + \Delta\phi_{\mathrm{R}}\right),$$

$$A_3\left(x = x_{\mathrm{res}} \ldots x_{\mathrm{max}}\right) = A_{\mathrm{T}} \mathrm{e}^{-\frac{x - x_{\mathrm{res}}}{l_{\mathrm{d}}}} \cos\left(2\pi \frac{x - x_{\mathrm{res}}}{\lambda_1} + \phi_{\mathrm{A}_1}\left(x_{\mathrm{res}}\right) + \Delta\phi_{\mathrm{T}}\right).$$

Here, l_{d} is the decay length of the λ_1 spin wave in YIG, ϕ_{in} is the phase offset of the excited wave, and $\Delta\phi_{\mathrm{in}}$ is the experimentally controlled phase offset (sweep parameter). To reproduce the experimental data, the phase change induced by reflection $\Delta\phi_{\mathrm{R}}$ and the phase change incurred by transmission $\Delta\phi_{\mathrm{T}}$ are used as fitting parameters. If no energy is absorbed within the resonator, one can write the amplitude of the reflected and transmitted spin waves in terms of the reflection R and transmission $T = 1 - R$ coefficients, and the incident wave amplitude at the resonator $A_{\mathrm{in}} \mathrm{e}^{-\frac{x_{\mathrm{Bi}}}{l_{\mathrm{d}}}}$. In this case, the wave amplitudes are given by $A_{\mathrm{R}} = R A_{\mathrm{in}} \mathrm{e}^{-\frac{x_{\mathrm{Bi}}}{l_{\mathrm{d}}}}$ and $A_{\mathrm{T}} = \left(1 - R\right) A_{\mathrm{in}} \mathrm{e}^{-\frac{x_{\mathrm{Bi}}}{l_{\mathrm{d}}}}$. The model allows for the inclusion of an absorption coefficient D, so that $1 = R + T + D$. However, the best fits of the TR-MOKE data are attained when D is set to zero or very close to zero, irrespective of frequency. This observation demonstrates low-loss spin-wave transport across the nanoscale resonator. **f** Derived transmission coefficient from fits to the TR-MOKE data as a function of frequency.

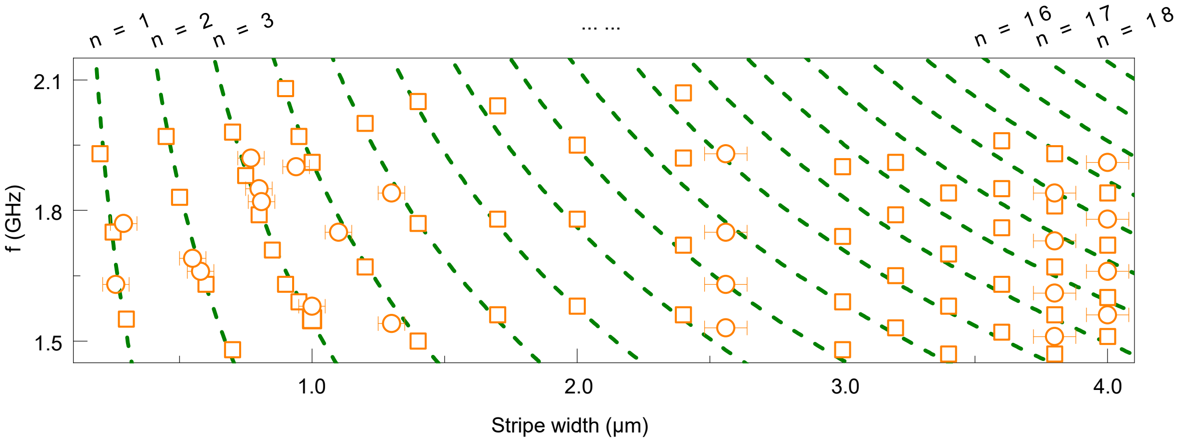

Supplementary Figure 9. Frequency of the spin-wave transmission gap as a function of CoFeB stripe width for $n = 1 - 18$. The green dashed lines are calculated using the condition of minimum transmission and the asymmetric dispersion relation of the YIG/CoFeB bilayer (squares in Fig. 3a). A constant value of $\varphi_0 = 1.34\pi$ is used for all transmission gaps. Experimental results (circles) and simulations (squares) of transmission gap frequencies agree with the magnonic resonator model.

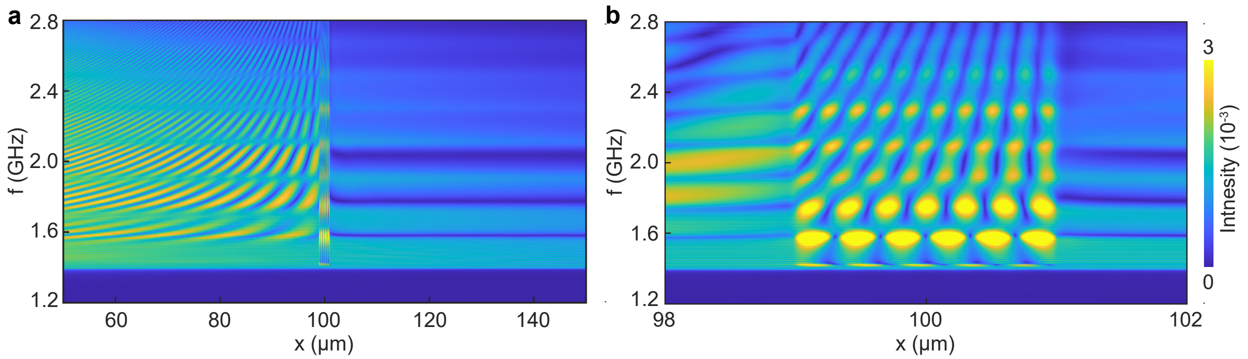

Supplementary Figure 10. **a** Simulated spatial distribution of spin-wave intensity in a 100-nm-thick YIG film with a 2-μm-wide CoFeB stripe centered at $x = 100$ μm. **b** Zoom-in of the YIG/CoFeB bilayer region. In the simulation, spin waves with wavelength λ_1 are excited continuously in the YIG film at $x = 0$. Inside the bilayer, the short-wavelength λ_3 mode and the λ_2 mode propagate along the $+x$ and $-x$ direction, respectively. Interference patterns at $x < 100$ μm illustrate strong spin-wave reflection from the bilayer region. As a result, transmission gaps form at $x > 100$ μm. Spin-wave interference conditions within the bilayer are affected by dynamic dipolar coupling between YIG and CoFeB. As a result, the frequency of minimum transmission (i.e. maximum reflection) does not exactly match the condition of minimum spin-wave intensity within the bilayer. As the dynamic dipolar coupling strength between the YIG film and the CoFeB stripe decreases for higher-order resonances, this discrepancy becomes smaller. The effect of dipolar coupling is taken into account by an additional phase shift in the Fabry-Pérot resonator model.

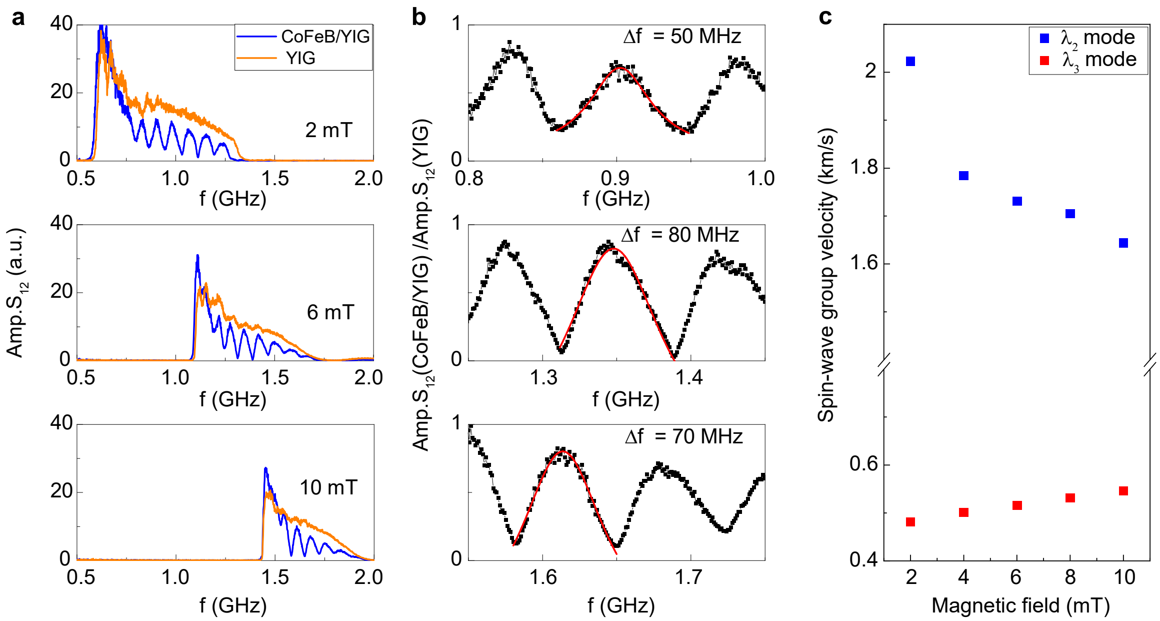

Supplementary Figure 11. **a** Spin-wave transmission spectra (amplitude of S_{12}) recorded on a 100-nm-thick YIG film with a 6-μm-wide CoFeB stripe (blue curves) and the same YIG film without stripes (orange curves) for $\mu_0 H_{\rm ext} = +2$ mT, $\mu_0 H_{\rm ext} = +6$ mT, and $\mu_0 H_{\rm ext} = +10$ mT. **b** Normalized spin-wave transmission spectra extracted from the data in (**a**). The red curves are Lorentzian fits to the resonance peaks between two transmission gaps. The Lorentziam linewidth Δf is indicated. **c** Group velocity of the λ_2 and λ_3 modes inside the resonator as a function of magnetic bias field. The data are derived from the spin-wave dispersion relation of the YIG/CoFeB bilayer.

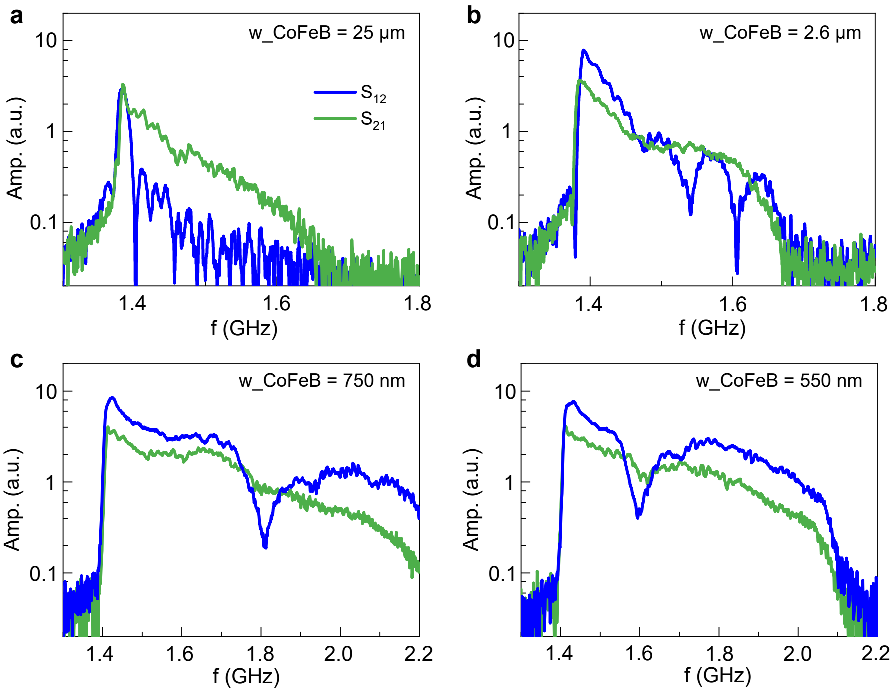

Supplementary Figure 12. **a-d** Spin-wave transmission spectra (amplitude of S_{12} and S_{21}) recorded on a 70-nm-thick YIG film with a 25-μm-wide CoFeB stripe (**a**), a 2.6-μm-wide CoFeB stripe (**b**), a 750-nm-wide CoFeB stripe (**c**), and four 550-nm-wide CoFeB stripes (**d**). $\mu_0 H_{\rm ext} = +10$ mT. Nonreciprocity of spin-wave transport (wide stripe) and transmission gaps (narrow stripes) are measured.

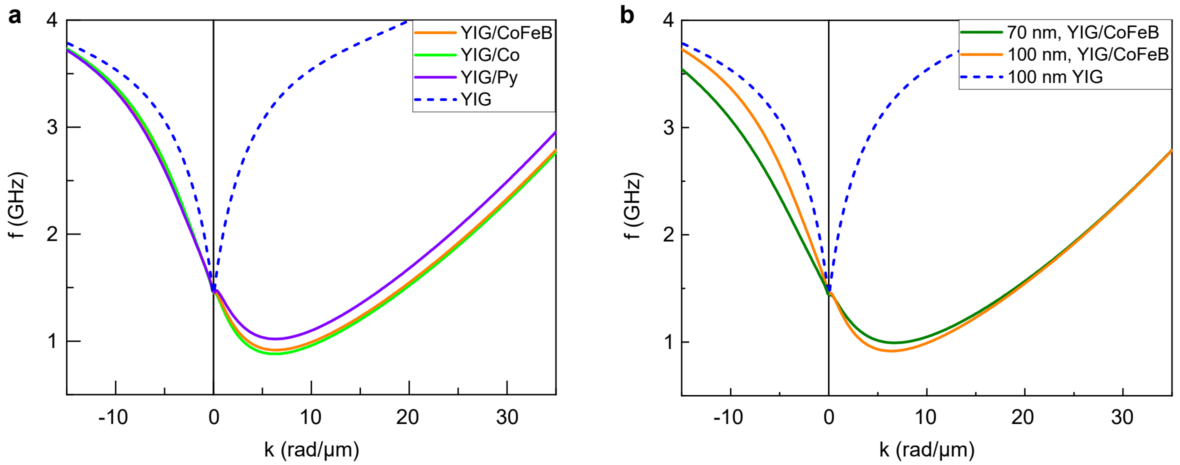

Supplementary Figure 13. **a** Spin-wave dispersion relations for a bare YIG film and YIG/Co, YIG/CoFeB, and YIG/Py bilayers. The YIG film is 100 nm thick and $\mu_0 H_{\mathrm{ext}} = +10$ mT. A reduction of the saturation magnetization ($M_{\mathrm{s,Co}} = 1.40 \times 10^6$ A/m, $M_{\mathrm{s,CoFeB}} = 1.15 \times 10^6$ A/m, $M_{\mathrm{s,Py}} = 0.80 \times 10^6$ A/m) results in a small up-shift of the dispersion curve for positive k. This effect is caused by a lowering of the dipolar coupling field. As a result, the spin-wave rejection frequencies of resonator structures comprising a continuous YIG film and a ferromagnetic metal stripe depend on the stripe material. **b** Dependence of the spin-wave dispersion relation on YIG film thickness. The dispersion curve of the YIG/CoFeB bilayer only changes slightly with decreasing film thickness. Consequently, strong nonreciprocity and the formation of transmission gaps persist in thin YIG films.

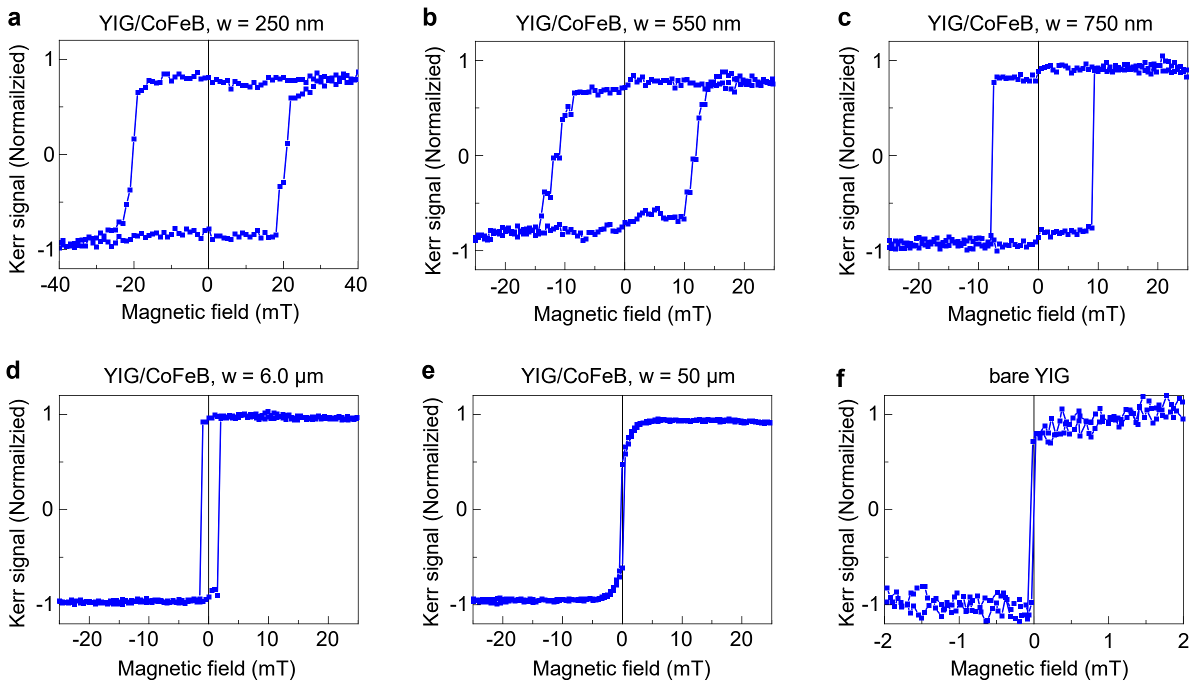

Supplementary Figure 14. **a-e** MOKE hysteresis loops of a YIG film with CoFeB stripes of different width (w = 250 nm (**a**), w = 550 nm (**b**), w = 750 nm (**c**), w = 6.0 μm (**d**), and w = 50 μm (**e**)). The measurements are performed using a MOKE microscope with the data collection window set to the YIG/CoFeB bilayer region. The signal is dominated by magnetic switching in the CoFeB stripe because its thickness prevents most light from transmitting to the YIG film. The switching field of the CoFeB stripe increases with decreasing stripe width. **f** MOKE hysteresis loop of an uncovered 100-nm-thick YIG film.

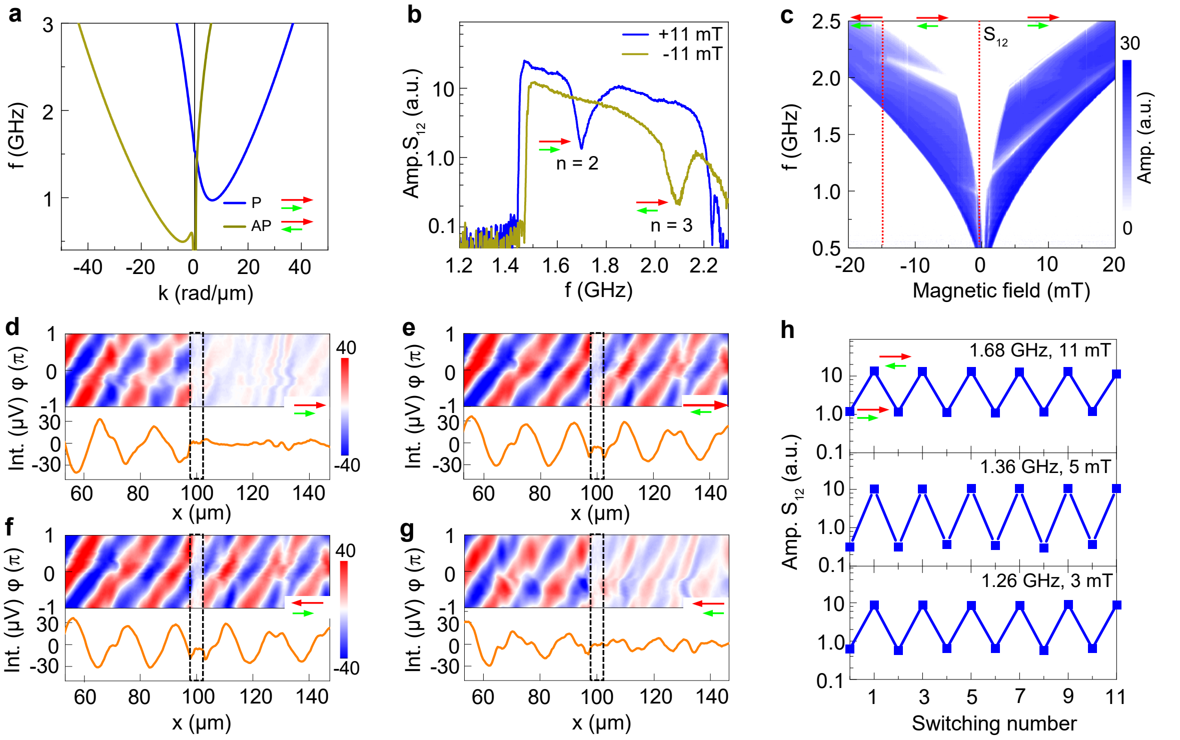

Supplementary Figure 15. **a** Spin-wave dispersion relations for a 100 nm YIG/50 nm CoFeB bilayer with parallel (P) and antiparallel (AP) magnetization configurations. **b** Spin-wave transmission spectra (amplitude of S_{12}) of a 100-nm-thick YIG film with four 550-nm-wide CoFeB stripes ($p = 1$ μm) for parallel (blue curve, $\mu_0 H_{\text{ext}} = +11$ mT) and antiparallel (dark yellow curve, $\mu_0 H_{\text{ext}} = -11$ mT) magnetization states. The magnetization of the YIG film is switched. The transmission gap for the parallel and antiparallel magnetization configuration correspond to the $n = 2$ and $n = 3$ resonance condition, respectively. **c** Contour plot of the S_{12} amplitude as a function of magnetic field for the same device structure. **d-g** Phase-resolved TR-MOKE microscopy maps and line profiles recorded at 1.68 GHz. Dashed lines indicate the region with four 550-nm-wide CoFeB stripes. **h** Modulation of the spin-wave transmission signal during sequential switching between parallel and antiparallel magnetization states. Data for three different bias fields are shown. In all panels, the red and green arrows depict the direction of magnetization in CoFeB and YIG, respectively.

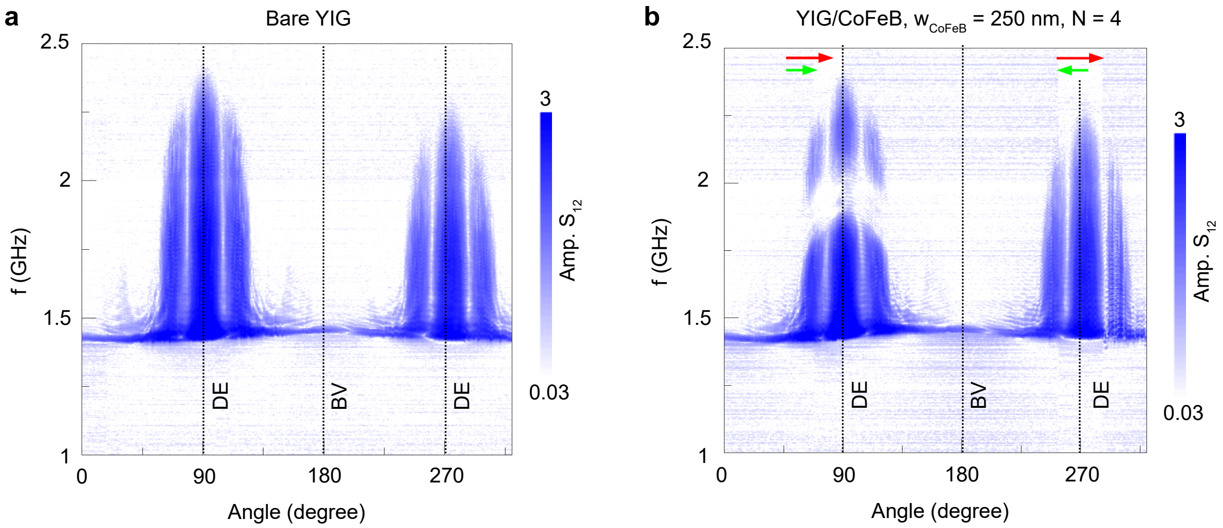

Supplementary Figure 16. **a,b** Contour plots of the S_{12} amplitude as a function of magnetic field angle for an uncovered 70-nm-thick YIG film (**a**) and the same YIG film with four 250-nm-wide CoFeB stripes ($p = 500$ nm) (**b**). The in-plane magnetic bias field is set to 10 mT. At this field strength, the magnetization of the CoFeB stripe remains fixed and the magnetization of the YIG films aligns along the bias field. The DE geometries with parallel and antiparallel magnetization alignment are indicated by arrows. The experimental data in **b** demonstrate continuous tuning of the transmission gap frequency when the magnetization of YIG rotates away from the DE configuration at 90° (parallel magnetization). The transmission gap for antiparallel magnetization is not visible because of its below-FMR frequency (see Fig. 5a in main manuscript). Because of inefficient spin-wave excitation by the microwave antenna, we do not measure a transmission signal when the angle between the wave vector and the magnetization of the YIG film is small, i.e., close to the backward-volume configuration.

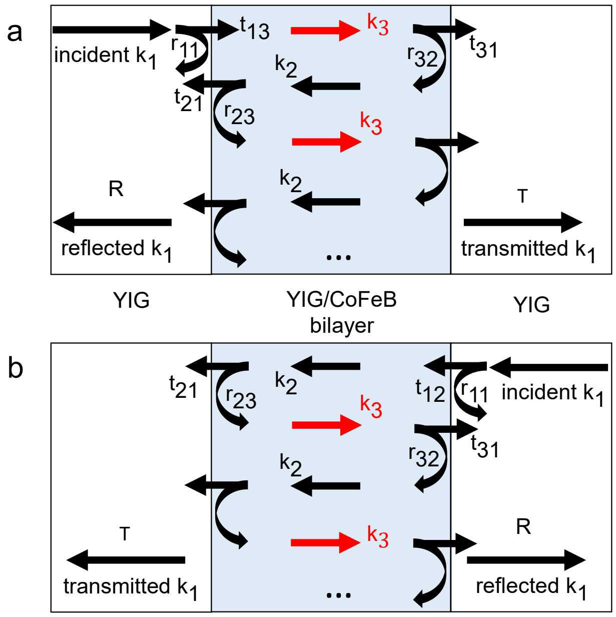

Supplementary Figure 17. **a,b** Schematics of the magnonic resonator with reflection and transmission coefficients for the geometry whereby the incoming λ_1 spin waves convert to the λ_3 mode (**a**) or λ_2 mode (**b**) at the first YIG/CoFeB interface.
